# EcoImprove: Revealing aquatic ecological effects of micropollutant discharge from municipal wastewater treatment plants

**DOI:** 10.1016/j.fmre.2022.09.034

**Published:** 2023-02-03

**Authors:** Yaohui Bai, Qiaojuan Wang, Hui Lin, Weiwei Ben, Zhimin Qiang, Huijuan Liu, Min Yang, Jiuhui Qu

**Affiliations:** a Key Laboratory of Drinking Water Science and Technology, Research Center for Eco-Environmental Sciences, Chinese Academy of Sciences, Beijing 100085, China; b University of Chinese Academy of Sciences, Beijing 100049, China; c Center for Water and Ecology, Tsinghua University, Beijing 100084, China

**Keywords:** Micropollutants, Aquatic ecological effects, Microbial community, Composition and function, EcoImprove project

## Abstract

Micropollutants (MPs) discharged from municipal wastewater treatment plants are of great environmental concern due to their toxicities to aquatic organisms. Given the knowledge gaps on how MPs affect receiving aquatic ecosystems, we initiated the EcoImprove project to unravel the causal relationship between MP discharge and variation in biocommunity (especially microbial community) composition and function in receiving aquatic ecosystems. After integrating laboratory studies, field investigations, and flume simulation experiments from 2014 to 2021, we investigated how different MPs affect the growth and metabolic function of microbial species, developed several microbial indicators to evaluate the effects of MP discharge on receiving rivers, and evaluated the ecological benefits of municipal wastewater treatment plant upgrade on receiving aquatic ecosystems. Here, we summarize the main outcomes of the EcoImprove project and propose future research plans to deepen our understanding of the ecological impacts of anthropogenic activity.

## 1 Introduction

Micropollutants (MPs) are compounds of potential toxicological concern that are continuously released from various sources, including wastewater effluent, and occur at low concentrations (ng/L to μg/L level) in natural environments [1]. MPs include pharmaceuticals and personal care products (PPCPs), endocrine disruptors, pesticides, and many other emerging compounds. As many of these compounds are designed to be active at low concentrations, their environmental toxicities should not be ignored. However, how MP discharge affects aquatic ecosystems remains poorly understood. To address this issue, we initiated the EcoImprove project (2014–2021) supported by the National Natural Science Foundation of China in cooperation with the Swiss Federal Institute of Science and Technology (Eawag). The main objectives of this project were to: (i) resolve how MPs from wastewater treatment plants (WWTPs) affect different biocommunities in receiving rivers in the context of other confounding factors such as nutrients; and (ii) explore how WWTP upgrade affects the ecological health of the receiving rivers. We conducted field surveys, simulation experiments, and laboratory experiments, integrating multidisciplinary techniques, including environmental chemistry, toxicology, ecology, and statistics. Here, we summarize the main scientific findings of the project and propose areas for future study.

## 2 Main outcomes

### 2.1 Bidirectional effects of MPs on microbial growth and function

**Fig. 1:**
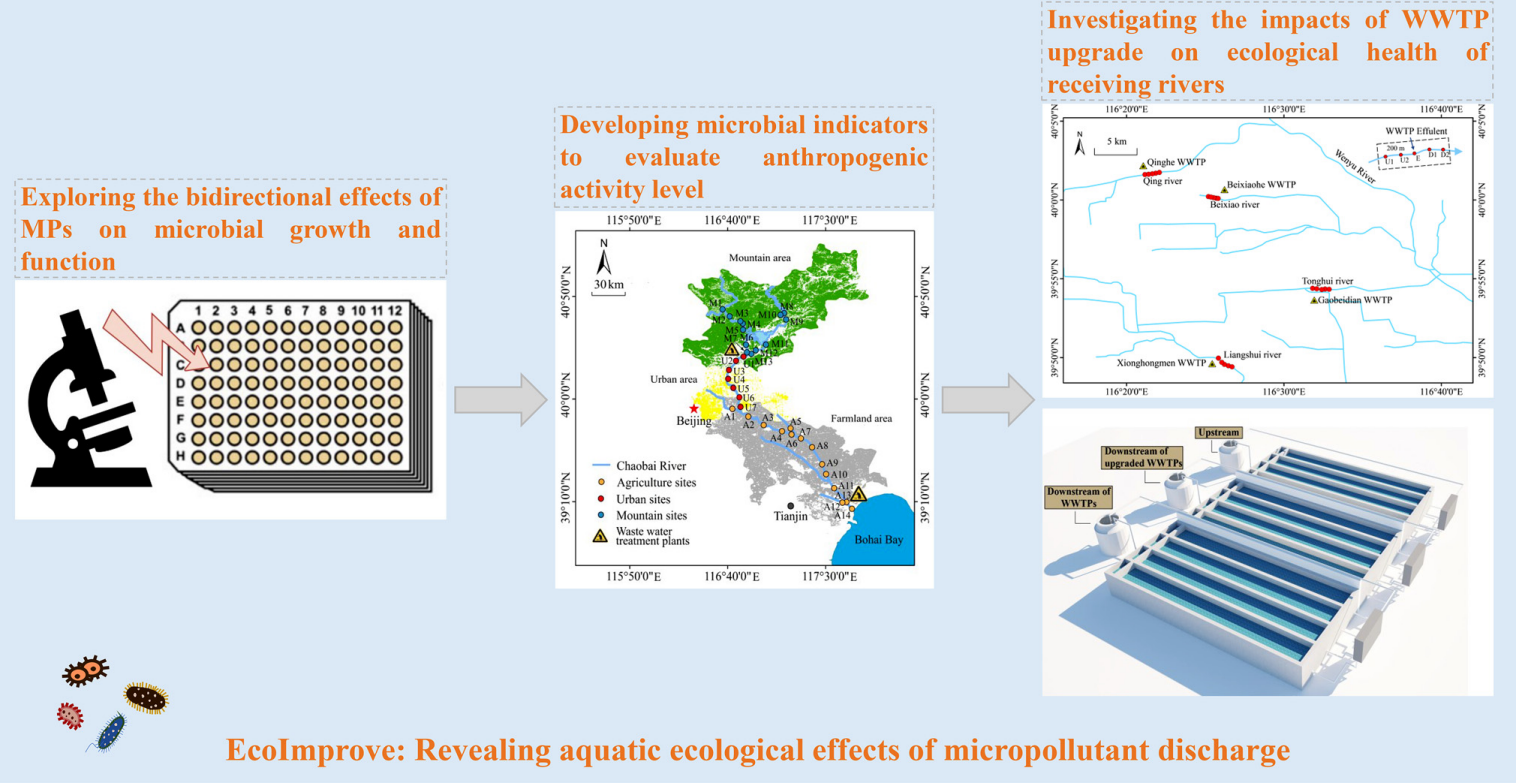
**Work-flow chart of EcoImprove project.** The project integrated laboratory experiments (left subfigure), field investigation (Chaobai River (middle) and four urban WWTP effluent-receiving rivers (upper right)), and flume experiment (lower right) to explore the aquatic ecological effects of micropollutant discharge.

Microbes are the most ubiquitous and abundant organisms worldwide. The wide-ranging interactions between MPs and microbes are essential for maintaining the self-purification of aquatic ecosystems. It was demonstrated that microbes can directly (biodegradation or co-metabolism of MPs) and indirectly transform MPs (formation of biogenic metal oxides and MP oxidation) [2]. In addition, MPs can negatively and positively (e.g., as growth substrates) affect microbial growth and function, and influence microbially controlled organic matter flux [3]. However, comprehensive information on the positive response of microbes to MP exposure is lacking as MPs are generally considered to be harmful to microbial growth and function in aquatic ecosystems. Thus, we first explore how different MPs affect the growth and function of single microbial strains in the lab (Fig. 1).

*Pseudomonas* is a type of bacteria that is found commonly in aquatic environments. Thus**,** we selected an Mn(II)-oxidizing bacterium *Pseudomonas* sp. QJX-1 using tyrosine as the sole carbon and nitrogen source to investigate the effects of seven MPs frequently detected in Beijing rivers (i.e., two UV filters (benzophenone-3 (BP-3) and BP-4), two pesticides (glyphosate and 2,4-dichlorophenoxyacetic acid), one additive (bisphenol A), one antibiotic (tetracycline), and one bactericide (triclosan)) on the growth and function of QJX-1. Results showed that six MPs inhibited bacterial growth and Mn(II) oxidation, whereas BP-4 (up to 5 mg/L) promoted bacterial growth and Mn(II) oxidation, although unexpectedly, its concentration was not directly coupled to growth. In addition, BP-4 accelerated tyrosine consumption by QJX-1, thereby accelerating the release of bacterial superoxide, which oxidizes Mn(II) to Mn(IV) [2].

*Comamonas* species are common environmental bacteria, and their abundance was found to be significantly changed in the Beijing rivers after WWTP upgrade. Therefore, we analyzed the response of *Comamonas testosteroni* CNB-2 to sulfonamide (frequently detected in rivers) exposure (unpublished). Results showed that the sulfonamides were inhibitory at high concentrations (above 1 mg/L) but stimulated cell proliferation and denitrification capability at low titers (from 5 μg/L to 250 μg/L). We demonstrated that the stimulation effect in cell proliferation and denitrification of *C. testosteroni* was associated with regulation of the *LuxR solos* protein, a transcriptional activator that can up-regulate the β-oxidation of fatty acids.

Overall, our laboratory experiments provide new evidence for how MPs may affect microbial growth and function and highlight the dual effects of MPs on aquatic microbes. Based on our observations and previous reports, we speculate that MPs generally inhibit microbial growth and function, but a small proportion promote certain microbial species in natural aquatic ecosystems. In addition, a recent study also found the micropollutants can enhance microbial succession with a replacement of more tolerant bacteria in water [4].

### 2.2 Planktonic microbial community as an indicator of anthropogenic activity

Microbes are sensitive to anthropogenic activities and may be potential indicators of activity levels. While many studies have documented the impacts of anthropogenic activity on changes in microbial communities, the direct use of microbial communities as indicators of such activity remains limited. Moreover, from culture and mesocosm experiments, we understand the microbial biomass and diversity are generally inhibited by micropollutants, but stimulated by nutrients in aquatic ecosystems. However, how to use microbial indicator to discern the respective effects of micropollutants and nutrients discharge on aquatic ecosystems is still unexplored.

To address this issue, we selected an anthropogenically disturbed river (Chaobai River, Beijing, China) with distinct land use partitioning (i.e., less disturbed mountainous area, urban area, and agricultural area) (Fig. 1) for field investigation. Results showed that: (i) pharmaceutical concentrations were highest in agricultural areas; and (ii) Caffeine, antibiotics and bezafibrate were identified as main risk driving compounds [5]. Consistent with the studies from Eawag, we also found the negative effects of MPs were “masked” by nutrient enrichment [6] and agriculture has more stronger impacts on the biological communities in the receiving aquatic ecosystems [7]. Subsequently, by combining water quality indices with planktonic microbial composition and function, we observed that anthropogenic activities promoted the production of bacteria, influenced the distribution of dominant species, and accelerated microbial metabolism of natural organic matter (NOM). After screening for possible factors affecting the microbial community, we developed a NOM metabolic index to quantitatively reflect the holistic influence of nutrients and MPs [8]. To further reflect the impacts of MPs on aquatic ecological health, we then developed a novel approach integrating metagenomic analysis and flow cytometry to identify and quantify potential pathogenic antibiotic-resistant bacteria (PARB), carrying both antibiotic resistance genes (ARGs) and virulence factor genes (VFs) in the aquatic environment, which are of concern due to their infectivity and antibiotic resistance. Based on PARB abundance/density, we confirmed that microbiological risk in samples from agricultural areas was significantly higher than that from urban areas. This may be due to the higher antibiotic use in agriculture as well as intragenomic ARG-VF co-evolution under antibiotic selective pressure [9].

Subsequently, we applied machine learning (mainly the random forest model) to predict water sample sources from three partitions of the Chaobai River based on environmental physicochemical indices (PCIs), microbiological indices (MBIs), and their combination. We observed that the MBI-based models exhibited several advantages over the PCI-based models and the combined PCI- and MBI-based models. Using abundances of the top 30 bacteria combined with PARB as input into model, we achieved a low median out-of-bag error rate (9.9%) and high median kappa coefficient (0.8694). Currently, machine learning is gaining great attention in predicting environmental phenomena from environmental microbial community data, especially pollution source apportionment [10]. Thus, with future improvement in sequencing technology and data availability, MBI-based models will provide an economical, rapid, and reliable way to identify the sources of water pollution [11].

### 2.3 Improvement in ecological health of receiving rivers after WWTP upgrade

In recent years, all major WWTPs in Beijing have been upgraded (based on cascade ozonation and membrane technologies) to improve water quality and restore aquatic species, as more than 90% of river water in the city is WWTP effluent. This has provided the opportunity to track changes in biological communities with water quality improvement in receiving rivers. We first investigated how water quality improvements have changed ecological function in receiving rivers. We selected four urban WWTP effluent-receiving rivers (Fig. 1) as study sites (two rivers with WWTPs undergoing upgrade during the sampling period and other two rivers with WWTPs previously upgraded for reference) to investigate changes in water quality and biological communities (planktonic microbes, invertebrates, benthic animals) across five years (2015–2019). We found that the concentrations of organic nitrogen and MPs in the receiving rivers decreased markedly after WWTP upgrade. Using 16S rRNA and shotgun metagenomic sequencing, it was demonstrated that WWTP upgrade decreased the abundance of nitrifying bacteria but increased the abundance of denitrifying and dissimilatory nitrate reduction to ammonium (DNRA) bacteria in the urban WWTP effluent-receiving rivers. The changes in N-metabolism-related bacteria may be an attempt to provide enough bioavailable N for planktonic growth, as ammonium conservation is enhanced in receiving rivers after WWTP upgrade [12]. Subsequently, we analyzed how WWTP upgrade affects health-related microorganisms in the receiving rivers. Using the microbiological risk method [9], we explored health-related microbes in the two rivers with WWTPs undergoing upgrade and one river with WWTPs previously upgraded. Results showed that WWTP upgrade significantly reduced the relative abundance of pathogens carrying ARGs and VFs. This was mainly attributed to the role of ozonation as a recent study showed that ozonation reduced a number of ecotoxicological effects of biologically treated wastewater by 66%-93% [13]. However, we identified *Pseudomonas* and *Aeromonas* as the dominant pathogenic taxa carrying functional VFs (e.g., mobility and offensive) in epilithic biofilms from the two rivers after WWTP upgrade [14], which may threaten both ecological and human health.

Finally, we explored whether WWTP upgrade restored biofilm community composition and function in the receiving rivers in the simulated flume reactors [15]. Results showed that the metabolic pattern and composition of the biofilm communities gradually recovered after nutrient and MP levels decreased. Overall, the above results highlight the importance of WWTP upgrade on the restoration of aquatic ecosystems.

## 3 Outlook

Through the EcoImprove project, we disentangled the causal relationship between MP discharge and community variation in aquatic ecosystems. Further, we will focus on the following issues.

### 3.1 Omics-based detection of water-borne pathogens

MP discharge accelerates the evolution and dissemination of water-borne pathogens. Traditional detection methods, including real-time polymerase chain reaction (RT-PCR) and culture-dependent techniques, cannot comprehensively evaluate the pathogenicity and environmental risk of water-borne pathogens. However, DNA- and RNA-based genomic techniques (e.g., metagenomics and metatranscriptomics) can be applied to study the abundance and risk-rank of pathogens and, more importantly, characterize pathogenic factors, which can reflect ecological health risks. Therefore, we need to develop an applicable omics-based methodology, including the construction of a reference database and bioinformatics protocols, to monitor water-borne pathogens in aquatic ecosystems.

### 3.2 Joint effects of disinfection and MPs on aquatic ecosystems

During the COVID-19 pandemic, large quantities of disinfectants and antibiotics were discharged into aquatic ecosystems from sewage treatment plants and surface runoff. Exposure to chlorine and antibiotics in aquatic ecosystems can result in changes in microbial community composition, increased microbial tolerance, and even functional changes in higher trophic level organisms [16]. To date, however, our understanding of the joint effects of disinfectants and MPs on aquatic ecosystems remains limited. Notably, does the presence of MPs and disinfectants improve the distribution and dissemination of pathogens? Do disinfectants accelerate the horizontal transfer of ARGs and VFs in the MP-contaminated aquatic ecosystem?

### 3.3 Raising social awareness

The extensive use of synthetic chemicals has positive effects on human welfare, but also poses risks to environmental health, especially the long-term disease burden of micropollutants in aquatic ecosystems. Hence, there is a trade-off between the benefits of these chemicals and the environmental pollution associated with their discharge [17]. Societal awareness of the environmental risks of using such chemicals and reducing their production and use are critical issues for achieving ecological civilization and sustainable development.

## Declaration of competing interests

The authors declare that they have no conflicts of interest in this work.

## References

[1] Schwarzenbach R.P., Escher B.I., Fenner K. (2006). The challenge of micropollutants in aquatic systems. Science.

[2] Chang Y.Y., Bai Y.H., Huo Y. (2018). Benzophenone-4 promotes the growth of a *Pseudomonas* sp. and biogenic oxidation of Mn(II). Environ. Sci. Technol..

[3] Azam F. (1998). Microbial control of oceanic carbon flux: The plot thickens. Science.

[4] Izabel-Shen D., Li S., Luo T. (2022). Repeated introduction of micropollutants enhances microbial succession despite stable degradation patterns. ISME Commun..

[5] Su D., Ben W.W., Strobel B.W. (2020). Occurrence, source estimation and risk assessment of pharmaceuticals in the Chaobai River characterized by adjacent land use. Sci. Total Environ..

[6] Burdon F.J., Bai Y., Reyes M. (2020). Stream microbial communities and ecosystem functioning show complex responses to multiple stressors in wastewater. Glob. Chang. Biol..

[7] Burdon F.J., Munz N.A., Reyes M. (2019). Agriculture versus wastewater pollution as drivers of macroinvertebrate community structure in streams. Sci. Total Environ..

[8] Liao K.L.L., Bai Y.H., Huo Y. (2018). Integrating microbial biomass, composition and function to discern the level of anthropogenic activity in a river ecosystem. Environ. Int..

[9] Liang J.S., Mao G.N., Yin X.L. (2020). Identification and quantification of bacterial genomes carrying antibiotic resistance genes and virulence factor genes for aquatic microbiological risk assessment. Water Res..

[10] Ghannam R.B., Techtmann S.M. (2021). Machine learning applications in microbial ecology, human microbiome studies, and environmental monitoring. Comput. Struct. Biotechnol. J..

[11] Wang C.C., Mao G.N., Liao K.L.L. (2021). Machine learning approach identifies water sample source based on microbial abundance. Water Res..

[12] Wang Q.J., Liang J.S., Zhao C. (2020). Wastewater treatment plant upgrade induces the receiving river retaining bioavailable nitrogen sources. Environ. Pollut..

[13] Kienle C., Werner I., Fischer S. (2022). Evaluation of a full-scale wastewater treatment plant with ozonation and different post-treatments using a broad range of in vitro and in vivo bioassays. Water Res..

[14] Mao G.N., Liang J.S., Wang Q.J. (2021). Epilithic biofilm as a reservoir for functional virulence factors in wastewater-dominant rivers after WWTP upgrade. J. Environ. Sci..

[15] Lin H., Wang Q.J., Zhou J. (2021). Recovery trajectories and community resilience of biofilms in receiving rivers after wastewater treatment plant upgrade. Environ. Res..

[16] Tandukar M., Oh S., Tezel U. (2013). Long-term exposure to benzalkonium chloride disinfectants results in change of microbial community structure and increased antimicrobial resistance. Environ. Sci. Technol..

[17] Eggen R.I.L., Hollender J., Joss A. (2014). Reducing the discharge of micropollutants in the aquatic environment: The benefits of upgrading wastewater treatment plants. Environ. Sci. Technol..

